# Supplementary material for: Association between oral microbiome and five types of respiratory infections: a two-sample Mendelian randomization study in east Asian population
Source: Front Microbiol. 2024 Apr 10;15:1392473. doi: 10.3389/fmicb.2024.1392473 (PMC11039966; doi:10.3389/fmicb.2024.1392473)
Supplement: Supplementary file 1 [file Data_Sheet_1.zip › Supplementary Materials/MR plots of saliva/Pneumonia/s__Centipeda_massiliensis_mgs_3300/leave_one_out.pdf]

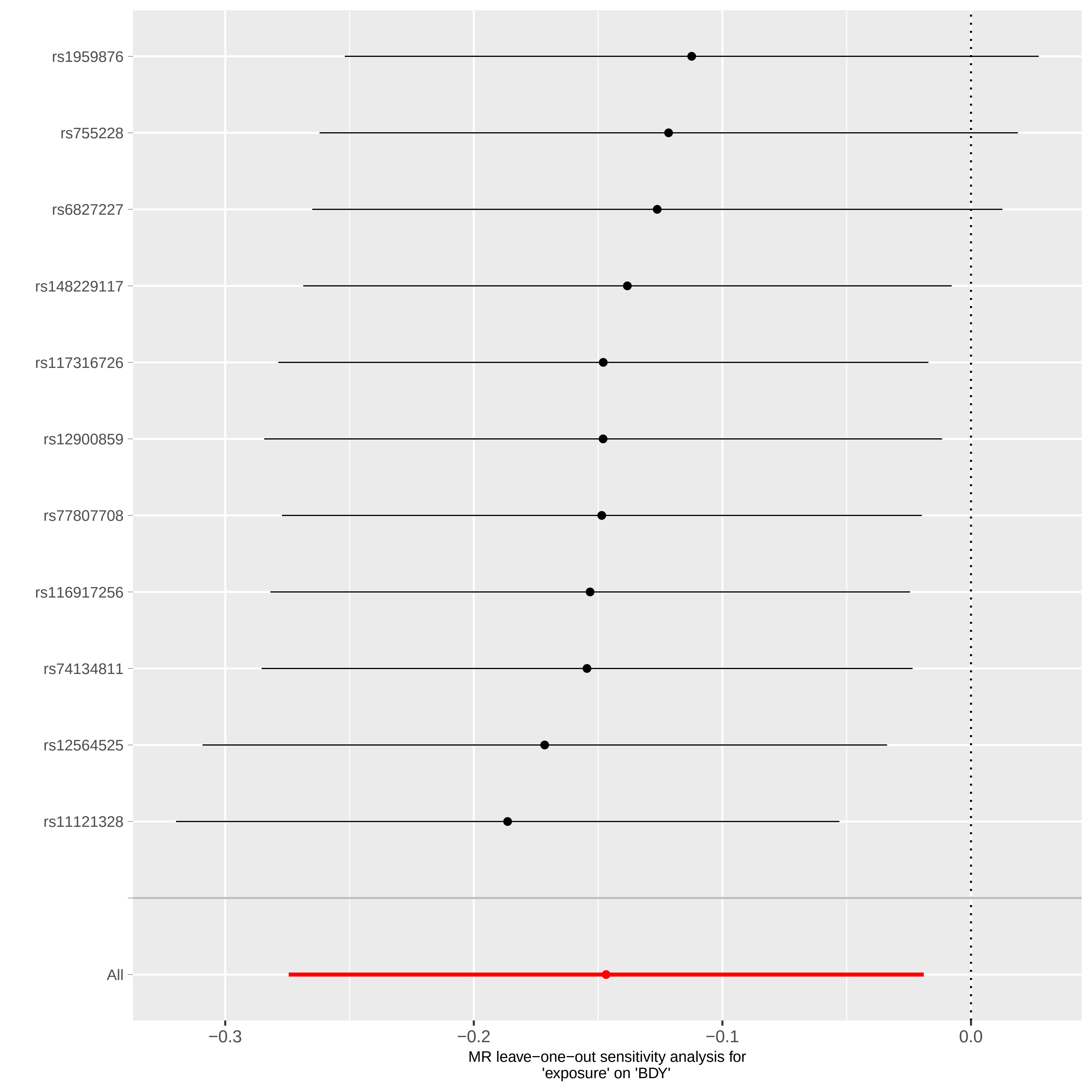
rs1959876
rs755228
rs6827227
rs148229117
rs117316726
rs12900859
rs77807708
rs116917256
rs74134811
rs12564525
rs11121328
All
−0.3
−0.2
−0.1
0.0
MR leave−one−out sensitivity analysis for
'exposure' on 'BDY'
